# Terbium Medical Radioisotope Production: Laser Resonance Ionization Scheme Development

**DOI:** 10.3389/fmed.2021.727557

**Published:** 2021-10-12

**Authors:** Vadim Maratovich Gadelshin, Roberto Formento Cavaier, Ferid Haddad, Reinhard Heinke, Thierry Stora, Dominik Studer, Felix Weber, Klaus Wendt

**Affiliations:** ^1^Institut für Physik, Johannes Gutenberg-Universität, Mainz, Germany; ^2^Institute of Physics and Technology, Ural Federal University, Yekaterinburg, Russia; ^3^Advanced Accelerator Applications, A Novartis Company, Origgio, Italy; ^4^GIP ARRONAX, Nantes, France; ^5^SY Department, CERN, Geneva, Switzerland; ^6^Institute for Nuclear and Radiation Physics, KU Leuven, Leuven, Belgium

**Keywords:** CERN-MEDICIS, RISIKO mass separator, terbium, gadolinium, laser resonance ionization, isotope separation, Ti:Sapphire laser, theranostics

## Abstract

Terbium (Tb) is a promising element for the theranostic approach in nuclear medicine. The new CERN-MEDICIS facility aims for production of its medical radioisotopes to support related R&D projects in biomedicine. The use of laser resonance ionization is essential to provide radioisotopic yields of highest quantity and quality, specifically regarding purity. This paper presents the results of preparation and characterization of a suitable two-step laser resonance ionization process for Tb. By resonance excitation via an auto-ionizing level, the high ionization efficiency of 53% was achieved. To simulate realistic production conditions for Tb radioisotopes, the influence of a surplus of Gd atoms, which is a typical target material for Tb generation, was considered, showing the necessity of radiochemical purification procedures before mass separation. Nevertheless, a 10-fold enhancement of the Tb ion beam using laser resonance ionization was observed even with Gd:Tb atomic ratio of 100:1.

## 1. Introduction

The novel CERN-MEDICIS facility aims for the production of non-conventional medical radioisotopes, being previously unavailable on the global market for biomedical research and development (1, 2). It is based on the use of electromagnetic mass separation for extraction of a desired radionuclide from a pre-irradiated target material (3). The facility exploits capabilities of the existing CERN accelerator complex around the ISOLDE on-line mass separator for radionuclide generation (4); in addition it is able to handle irradiated materials from other nuclear facilities for further extraction and purification of radioisotopes (5, 6).

The main application fields for medical radioisotopes are diagnostics and therapy, notably for cancer treatment (7). Both are accompanying each other, and the correctness and precision of the former defines the effectiveness of the latter. Moreover, in a personalized treatment modality, the use of the theranostic approach is rapidly developing: diagnostic and therapeutic active agents should imply radionuclides of the same chemical element or at least those species or compounds having very similar chemical properties, to reach the full control of the radiopharmaceuticals performance in the organism (8).

One promising chemical element for nuclear medicine and specifically for the theranostic approach is terbium. This lanthanide element, entitled as “Swiss army knife” of nuclear medicine (9), offers four different radioisotopes to researchers providing a desired combination of α or β^−^ therapy (^149^Tb, ^161^Tb) along with PET or SPECT imaging (^152^Tb, ^155^Tb) (10–15). Whereas ^161^Tb can be produced in nuclear reactors, the other three isotopes are available only at cyclotrons (16) or at radioactive ion beam facilities, like CERN-MEDICIS (17). Unfortunately, their production process is associated with high isobaric and isotopic contaminations, and, therefore, their use is not yet well-established (18).

In 2019, the dedicated laser ion source MELISSA was implemented to improve the production yield of the CERN-MEDICIS mass separator (19). Via a laser based multi-step excitation and ionization process, ideally only a single chemical element of interest is selectively ionized by laser photons, which are fine-tuned to resonances of strong transitions between atomic energy levels of the element (20). Due to the high efficiency of the laser ionization process via transitions into auto-ionizing levels, the ion beam production, and extraction throughput are strongly enhanced, while the product purity against other elements is in parallel improved due to the high selectivity of the resonance ionization technique (21, 22). In combination with electromagnetic mass separation, the laser ion source thus provides an output, which is almost entirely free of any kind of isobaric or isotopic contaminations (23).

Nevertheless, the use of laser resonance ionization for isotope separation requires the identification and characterization of the most suitable laser excitation scheme for later routine application. In this work, a highly efficient two-step ionization of terbium was investigated as optimum and simple for robust technical implementation. The efficiency and selectivity of the scheme was examined at the Mainz RISIKO mass separator, which offers very similar ion source conditions as the CERN-MEDICIS facility.

## 2. Materials and Methods

### 2.1. Experimental Setup

**Figure 1:**
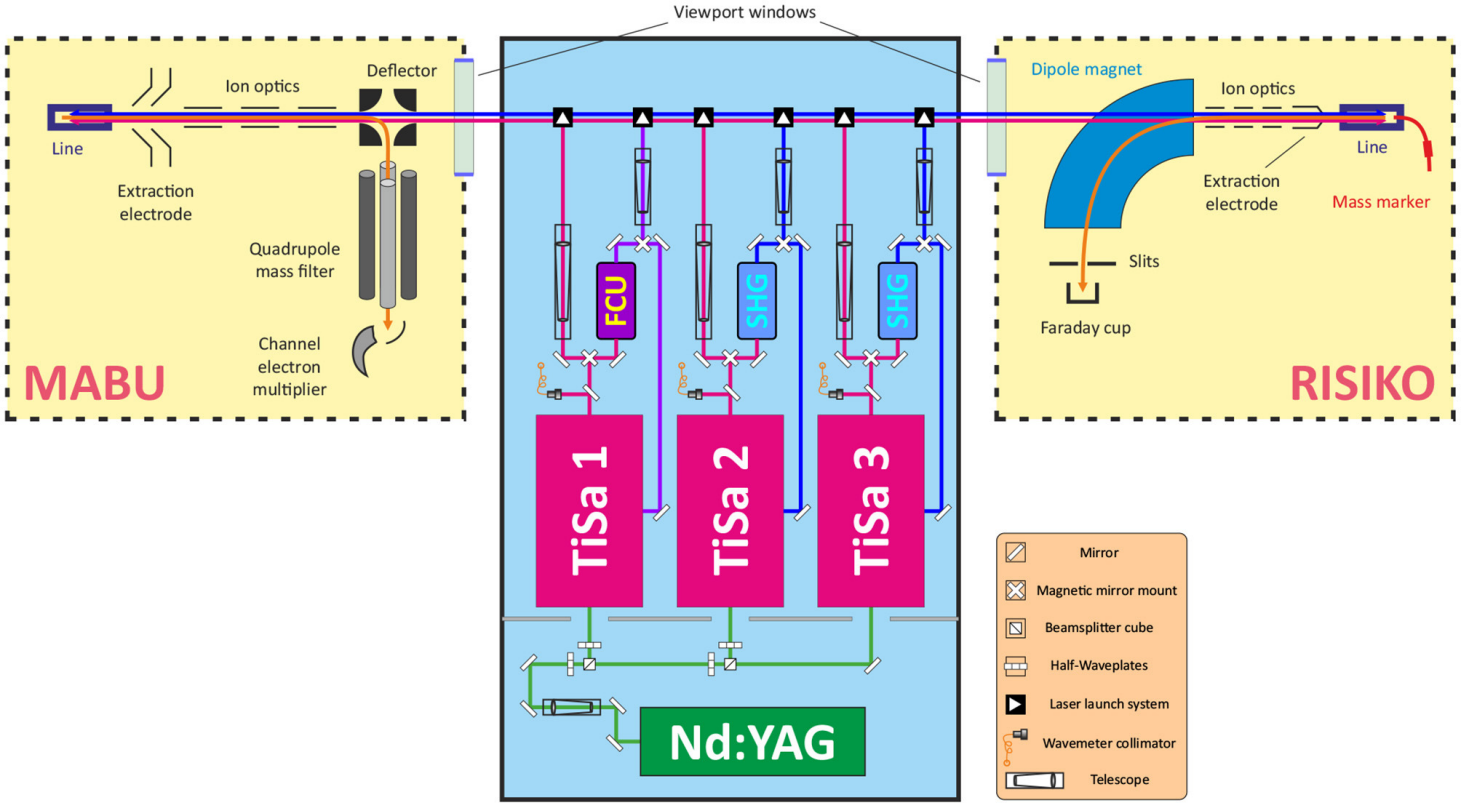
Schematic layout of MABU mass spectrometer, RISIKO mass separator, and a dedicated laser system (not to scale). Green, red, and blue lines represent corresponding laser beams; orange line—ion beam.

The LARISSA team of Mainz University operates two specific experimental setups for atomic spectroscopy and R&D on resonance ionization and isotope separation. The Mainz Atomic Beam Unit (MABU) is a compact quadrupole mass spectrometer (24), well-suited for spectroscopic analysis, which was used in this work to develop a suitable laser ionization scheme for Tb. The RISIKO setup (23) is a 30 keV beam energy electromagnetic off-line mass separator (in the experiment, the mass resolution was about 150 for the region of Tb isotopes), which serves as a substitute of the CERN-MEDICIS mass separator due to their widely similar experimental arrangement. RISIKO was used to perform ionization efficiency measurements and to study the ion source performance under the presence of contaminants, simulating the real production conditions of Tb radioisotopes. Both setups involve a dedicated laser system to induce the laser ionization process (see Figure 1).

One of crucial components of both setups is the ion source. For MABU it is a 35 mm long ionization tube, made of tantalum with inner and external diameters of, respectively, 2.5 and 4.5 mm, also called “hot cavity” or “line.” The RISIKO ion source also consists of this hot cavity, and it is equipped with an additional sample reservoir (or “mass marker”)—a thin 80 mm long capillary made of tantalum with inner and external diameters of 1.1 and 2 mm, respectively, which is directly connected to the ionization tube. A sample under investigation can be placed either in the ionization tube or in the sample reservoir. The line and the mass marker independently can be resistively heated up to 2,200°C to atomize and ionize the sample material via thermal surface ionization (the temperature distribution is not uniform, and derived from the comparison of simulation and direct measurements with pyrometer) (25). Together they represent an analogue of the target unit of CERN-MEDICIS mass separator: the ionization tube is almost identical and the sample reservoir represents the target container, from which radioactive nuclides effuse to the hot cavity through a transfer line (3).

To induce the stepwise resonance ionization process, laser beams are guided through a viewport window into the vacuum chamber and travel anticollinearly to the ion beam into the hot cavity to interact with the sample material and to form laser ions After extraction from the hot cavity, ion optics provide appropriate ion beam shaping. Mass separation of the ion beam is performed by the quadrupole mass filter of MABU or alternatively by the dipole magnet at RISIKO. As detector, a channel electron multiplier at MABU (for sensitive single ion detection) is used, or a Faraday cup at RISIKO (for quantitative ion current measurement), shielded with the guard voltage of 100 V to suppress the undesired contribution to the signal from secondary electrons. During all experiments, laser parameters (working wavelengths, position, timing, output power) are carefully controlled, recorded and readjusted to keep maximum ion signal. Lasers are usually set to maximum achievable output power, measured before the viewport window, and are overlapped spatially and temporally with a focus in the hot cavity.

The laser system consists of a number of solid-state lasers, which allow to implement and rapidly vary a multi-step resonance ionization process for chosen elements. A commercial Nd:YAG laser (Photonics Industries DM60, 532 nm with a repetition rate of 10 kHz) is used to pump up to three Ti:Sapphire lasers of the Mainz University design (26). With 15 W of pump power, a Ti:Sapphire laser generates emission in a wide-tunable wavelength range of 690–1,000 nm with ~40 ns long pulses and average output power up to 5 W, with typical 3–5 GHz spectral linewidth (27), resembling the MELISSA laser system at CERN-MEDICIS (19).

### 2.2. Spectroscopic Investigations

Laser resonance ionization spectroscopy on Tb atoms was performed in the past to study its high-lying and auto-ionizing states (28). Three-step laser ionization schemes were identified based on the wavelength range of dye lasers with first excitation transitions of 510 or 602 nm. These wavelengths are far off the working range of Ti:Sapphire laser, and therefore are not directly applicable for CERN-MEDICIS.

**Figure 2:**
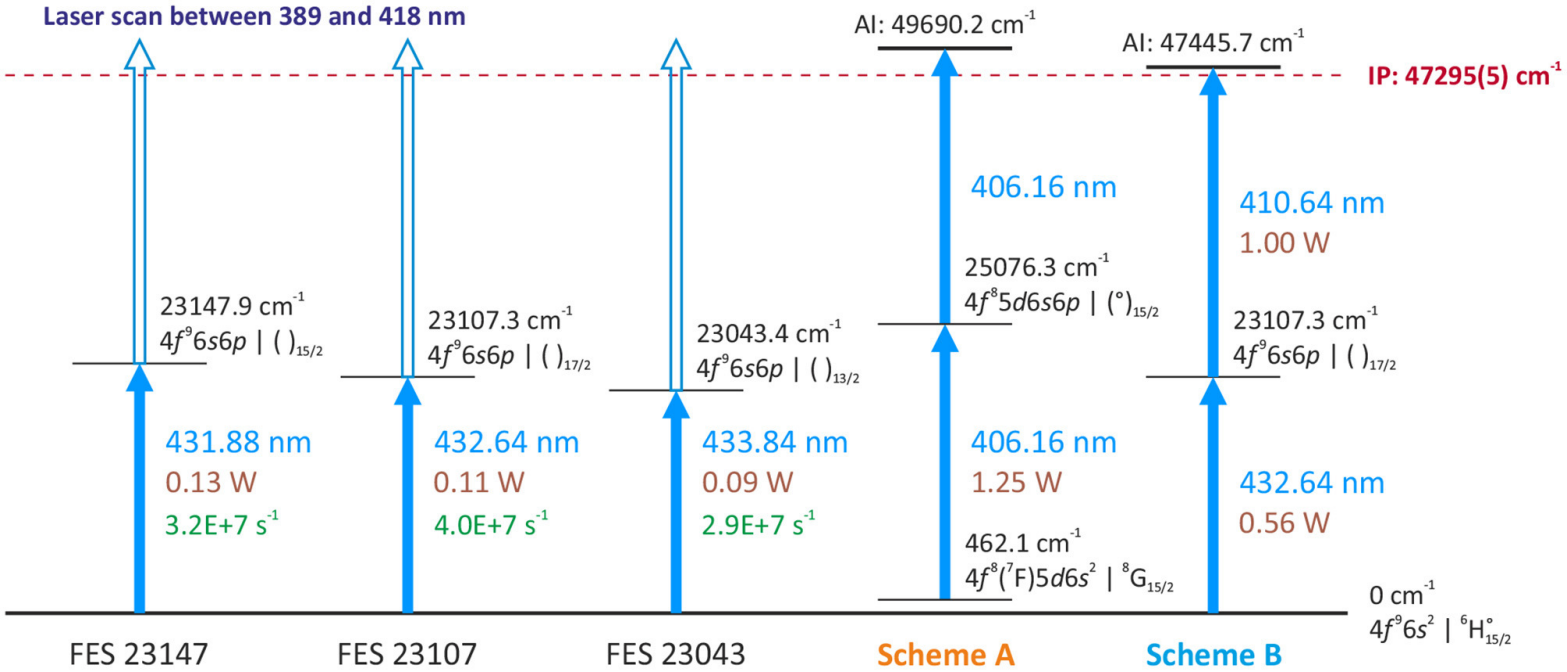
Overview of investigated first excitation steps (FES) and laser resonance ionization schemes, used for efficiency measurements.

Three excited energy levels at 23147.9, 23107.3, 23043.4 cm^−1^ (29) were addressed from the ground state by the second harmonic of a Ti:Sapphire laser in (30), as a basis for a three-step ionization process. Nevertheless, from these first excitation steps (FES) it is possible by using Ti:Sapphire second harmonic output to ionize Tb atoms with a direct further step, either via a transition into the ionization continuum or alternatively via an auto-ionizing state located slightly above the first ionization potential (see Figure 2). Since recently a spectroscopic scan in this spectral region is possible thanks to an automated grating-tuned Ti:Sapphire laser design, which was not available few years ago (31, 32). To characterize these second excitation steps (SES) into different auto-ionizing levels and to develop a suitable laser ionization scheme, the resonance ionization spectroscopy was undertaken at the MABU mass spectrometer using a pure Tb sample, made of AAS standard solution^1^.

### 2.3. Efficiency Measurement

A crucial performance characteristic of a newly developed scheme is its ionization efficiency. Commonly, the efficiency of a separation process can be measured as the ratio between the total number of detected ions *N*_*det*_ against the initial number of atoms in the sample *N*_*sample*_ (33). To evaluate the laser performance in the process ϵ_*laser*_, it is necessary to calculate the difference between efficiencies with and without lasers under the same experimental condition, to omit other factors of the process (34).

Therefore, the laser ionization efficiency can be calculated using the formula


(1) $$\begin{array}{r} \epsilon_{laser}=\frac{N_{det}-N_{surf}}{N_{sample}} \end{array}$$

where *N*_*surf*_ is the ion signal determined with blocked lasers. Namely, this number of ions reflects the contribution of thermally ionized atoms from the surface ionization. This contribution can be estimated during the measurement by periodically blocking the laser beams (35).

**Figure 3:**
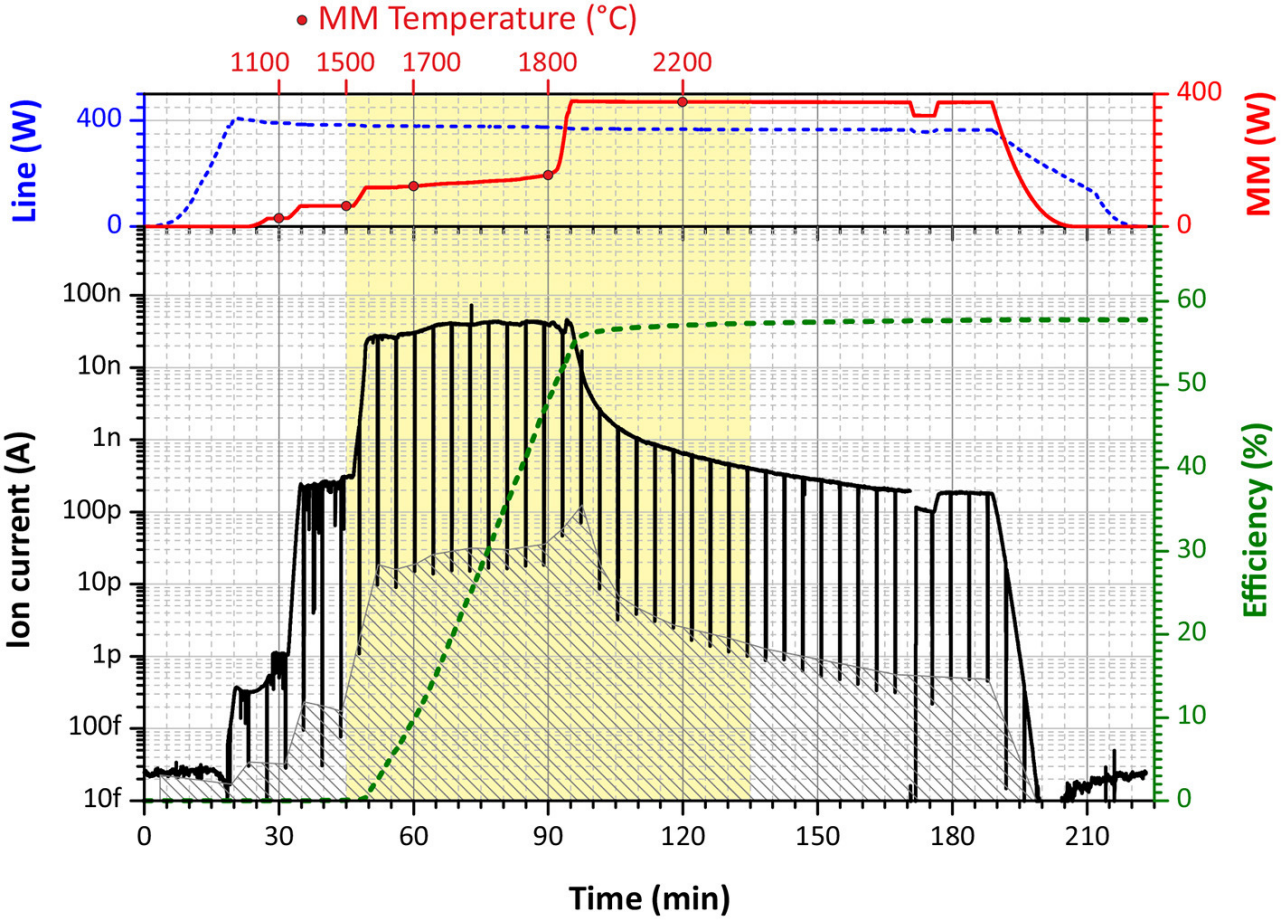
A graphical representation of a typical ionization efficiency measurement (in this case, scheme B, run 4). **(Upper Panel)** The blue dashed curve—the heating power applied to the ionization tube (“Line”), the red solid curve—the heating power applied to the mass marker (“MM”) with circle dots for specific temperature marks. **(Lower Panel)** The black solid curve—the measured ion current on Faraday cup, the gray filled area—the interpolated background signal of surface ionization (laser blocked), the green dashed curve—cumulative overall ionization efficiency.

All efficiency measurements were accomplished interlaced with a blank sample measurement before each new run of a Tb sample (the same procedure for both). That is necessary to ensure the absence of remaining Tb atoms either in the ionization tube or in the sample reservoir, which can bias final results as memory effect. A typical efficiency measurement is presented in Figure 3. The ion current is monitored on mass 159 u, corresponding to the only stable Tb isotope. During the first 40–50 min, the RISIKO mass separator is set to the working parameters; the hot cavity is heated up to 2,100°C (“Line power” of 400 W); the sample reservoir temperature is increased stepwise up to 1,500°C (in the Tb case), accompanied by the optimization of the mass separator and laser fine adjustments onto the maximum of the ion signal. In the next 45 min most Tb atoms are extracted by a smooth increase of the sample reservoir temperature up to 1,800°C; this period gives the highest contribution to the measured efficiency value; the surface ion signal is monitored each 4 min, blocking laser beams for 10 s (vertical black stripes in Figure 3). Afterwards, the ion signal is decreasing, and the sample reservoir temperature is pushed to the maximum of 2,200°C to extract the residual Tb atoms down to a negligible level; this last step is also useful to avoid contaminations in further measurements. During all measurements, the average laser power for the first excitation step was 0.56 W, and for the second excitation step about 1 W.

Each efficiency measurement was accomplished with a pure Tb sample, prepared by diluting of AAS standard solution^2^ of stable terbium oxide (Tb_4_O_7_; 1,000 μg/ml Tb concentration) with deionized water in the rate of 1–10. Five microliters (1.14 × 10^15^ Tb atoms) of the diluted mixture was pipetted onto a Zr 4 × 4 mm^2^ carrier foil of 25 μm thickness, serving also as a reducing agent, which was afterwards folded to fully enclose the dried sample. The uncertainty of the pipetting procedure was estimated to be around 3–5% (36), and it is the main source of imprecision for an individual efficiency measurement (35).

### 2.4. Laser Ion Source Performance Using a Gd Surplus Sample

The typical problem of medical radioisotope production is the presence of isotopic contaminants, in unfavorable cases in orders of magnitude higher than the desired radionuclide, which cannot be removed just by means of chemical separation. This problem can be solved by mass separation. While a surface ion source is not selective against isobaric contaminants with similar chemical properties and thermal ionization behavior, the combination of laser resonance ionization and electromagnetic isotope separation allows to diminish or even overcome these difficulties (23). Nevertheless, electromagnetic mass separation has a limited resolution: the tails of neighboring mass peaks can strongly interfere on the desired mass, particularly, if the quantity of interfering material is very high. As a result, the final product can be contaminated, and may require additional post-purification procedures in order to meet purity standards (leading to time-related and efficiency-related losses of radioisotopes). For very severe contaminations within the ion source, even the separation efficiency can be significantly decreased due to disturbed performance of the laser ion source (37).

For the cyclotron-based production route of terbium radioisotopes, a typical target material is natural or isotopically-enriched gadolinium (38). They both are lanthanides and have almost identical thermal ionization efficiency. In case of Tb-152 or Tb-155 generation, isobaric Gd isotopes will be considered as dominant contaminants for the product. For instance, after irradiation of natural Gd target at the ARRONAX cyclotron, the typical concentration of generated Tb-155 radioisotope is estimated about 1 ppm (i.e., Gd:Tb ratio of 10^6^:1). To determine an appropriate limit for before-separation radiochemistry procedures and to assess the performance of the separation process in this situation, the developed ionization scheme was tested with tailored samples, simulating the extraction of Tb isotopes in the presence of Gd target material. For this purpose, two sample mixtures were prepared using AAS standard solutions of stable terbium oxide (Tb_4_O_7_)^3^ and stable gadolinium oxide (Gd_2_O_3_)^4^: one with 50:1 and another with 100:1 atomic ratio of natural Gd to Tb (for both samples, the Tb quantity was the same as for efficiency measurements, i.e., 1.14 × 10^15^ atoms), which assumingly might already affect the Tb extraction. The behavior of the ion signal under different sample reservoir temperatures for Tb and Gd isotopes was characterized with and without laser beams.

## 3. Results

### 3.1. Resonance Ionization Spectroscopy

**Figure 4:**
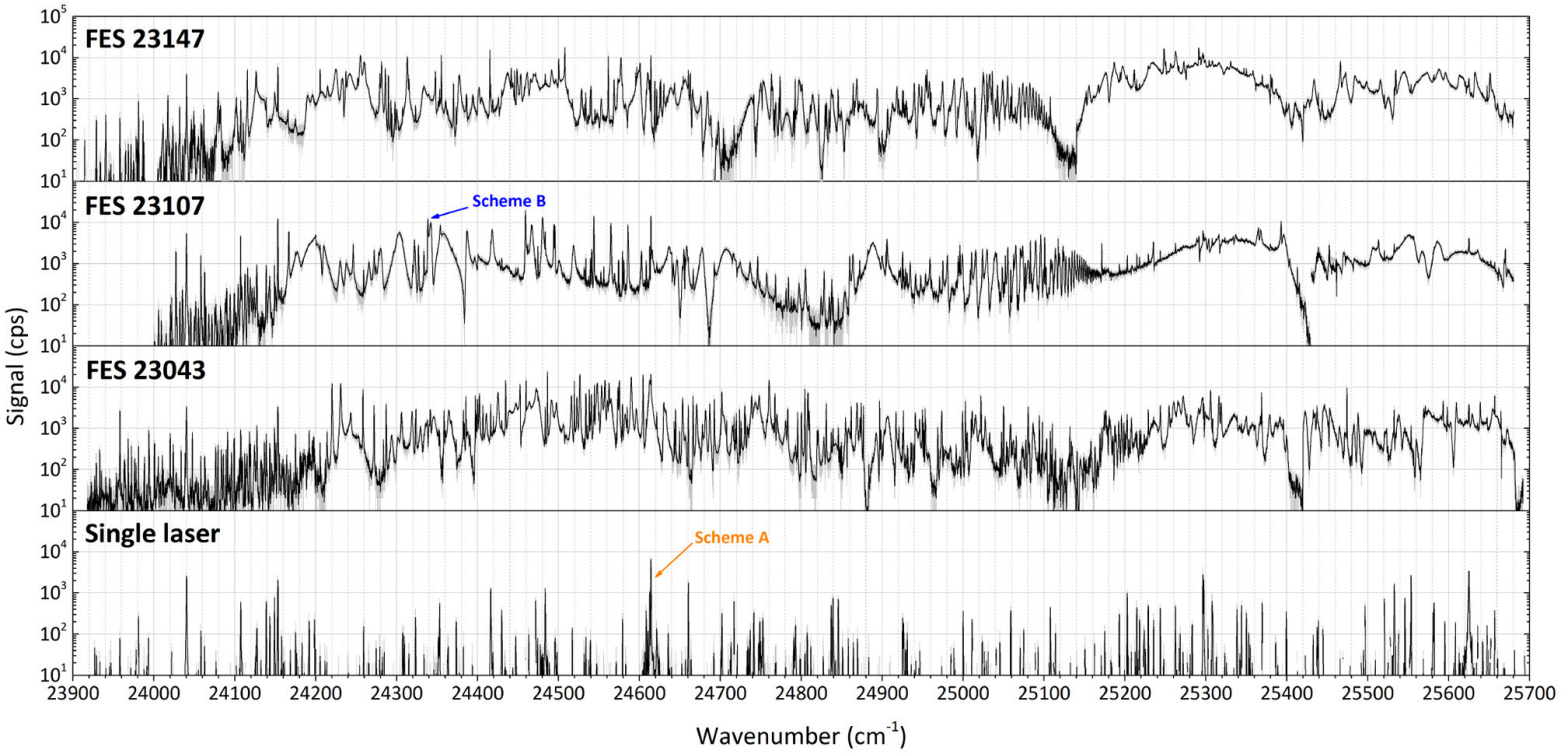
Laser resonance ionization spectra of terbium for scans of the second excitation step (SES) in three different two-step photoionization schemes: starting from first excitation steps (FES) of 23147.9, 23107.3, and 23043.4 cm^−1^. At the bottom the spectrum of a scan using just one laser in the considered region is given (schemes A and B, see text).

Three different first excitation steps, FES 23147, FES 23107, FES 23043 (see Figure 2), were used to develop an optimum two-step resonance ionization process for terbium. The merged results of series of spectroscopic investigations for a second excitation step are presented in Figure 4, exhibiting a very rich spectrum of observed auto-ionizing states. Coincidentally, it was observed that a spectrum of laser-dependent resonances can be obtained using only one laser (see the bottom spectrum in Figure 4); some of these resonances belong to so-called two-step single-color laser resonance ionization processes, where the respective wavelengths required for first excitation step and subsequent second ionization transition overlap within the laser linewidth.

Comparing the multitude of observed laser combinations under the same conditions (during the spectroscopy the laser power could not be kept constant), two ionization schemes with relatively higher ion signals among all the others of similar type (Figure 4) were chosen to characterize their performance: the single-color scheme A [starting from a thermally populated level at 462.1 cm^−1^ (29)] and the two-color scheme B (from the ground state, see Figure 2). Their positions in the spectra are indicated in Figure 4. Both schemes seem promising to ensure a highly efficient ionization process. Despite a slightly lower signal for scheme A, it is convenient for many applications to resonantly ionize Tb atoms only with one laser; therefore it was also taken into consideration. More information on the spectroscopic measurement results and the explanation on scheme choice can be found in the Supplementary Materials to this article.

### 3.2. Efficiency Measurement Results

**Figure 5:**
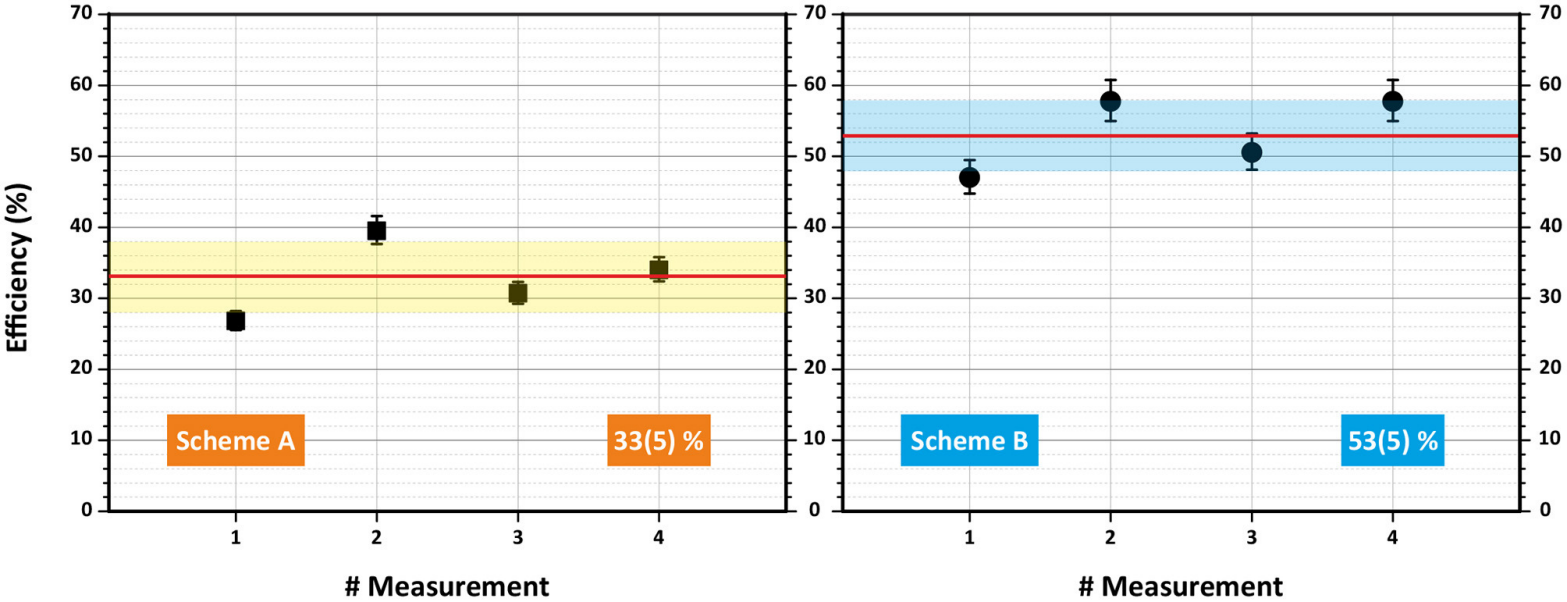
Overview of laser resonance ionization efficiency measurements for terbium for schemes A **(left)** and B **(right)**. Mean value and standard deviation are indicated.

The data obtained from each efficiency measurement run for both schemes are presented in Table 1. Graphically, an overview of results is visualized in Figure 5. The error bars for each individual run correspond to the uncertainty of the pipetting procedure (5% less or more atoms in the sample). One can see that all measurements were clearly reproducible. The single-color scheme A demonstrated a good average ionization efficiency of 33% with standard deviation <5%. The two-color scheme B showed an excellent performance with 53% average ionization efficiency also with <5% standard deviation.

**Table 1:** Experimental results of Tb laser resonance ionization efficiency measurements for schemes A and B (sample size is 1.14 × 10^15^ atoms).

**Scheme/**	**Detected ions**	**Detected ions**	**Background ions**	**Laser ionization**
**Run**	**from blank sample**	**from Tb sample**	**from Tb sample**	**efficiency**
	**(laser ON)**	**(laser ON)**	**(laser OFF)**	**%**
A/1	6.01 × 10^12^	3.10 × 10^14^	6.23 × 10^12^	(26.8 ± 1.3)
A/2	8.49 × 10^12^	4.49 × 10^14^	0.50 × 10^12^	(39.5 ± 1.9)
A/3	0.27 × 10^12^	3.50 × 10^14^	1.30 × 10^12^	(30.7 ± 1.5)
A/4	0.60 × 10^12^	3.87 × 10^14^	1.39 × 10^12^	(34.0 ± 1.6)
B/1	0.12 × 10^12^	5.36 × 10^14^	2.34 × 10^12^	(47.0 ± 2.2)
B/2	0.19 × 10^12^	6.57 × 10^14^	1.30 × 10^12^	(57.7 ± 2.8)
B/3	0.01 × 10^11^	5.75 × 10^14^	0.78 × 10^12^	(50.6 ± 2.4)
B/4	0.27 × 10^12^	6.56 × 10^14^	0.79 × 10^12^	(57.7 ± 2.8)

One can notice that the total laser enhancement factor (the ratio between detected ions with and without lasers) for almost all runs ranged between 500 and 1,000 compared to the surface ionization contribution without lasers. Except for the first two measurements (A/1 and A/2), the blank sample tests verified a negligible level of remaining Tb atoms in the ion source of <0.1% of the initial sample before the efficiency measurement. The total detected ions for a blank sample were at least twice less than the thermally ionized background, obtained for a real sample with blocked lasers. This eliminates the doubts, whether the efficiency measurements are interfered between each other.

**Figure 6:**
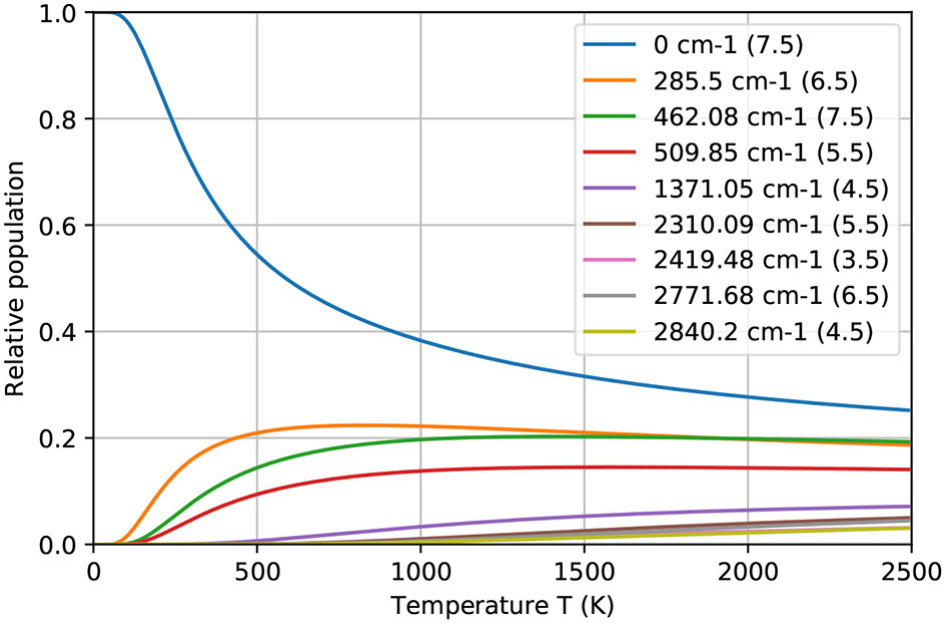
The relative population of ground and thermally excited states of Tb atom at different temperatures, according to the Boltzmann distribution. In legend the total angular momentum J for corresponding energy level is indicated.

A scrupulous reader can remark that the obtained ionization efficiency values clearly exceed the statistical thermal population for both starting energy levels of the investigated ionization schemes in the working temperature range (see Figure 6). This fact can be explained as follows: during the excitation or “depopulation” process of a starting level, thermal equilibrium is disturbed. Afterwards, atoms from other levels are de-excited or “re-populated” to the starting level to establish again the equilibrium. In order to achieve these high ionization efficiency values, the residence time of a Tb atom in the hot cavity has to be long enough to allow for re-population of the starting level over the course of multiple laser pulses. The RISIKO ion source geometry facilitates this process by requiring a high number of wall collisions before the atom exits, especially due to the narrow capillary of the sample reservoir, which is at first accessible for laser irradiation. Also, the low vapor pressure of Tb implies a longer time for wall sticking, promoting equilibration.

### 3.3. Laser Ion Source Performance

**Figure 7:**
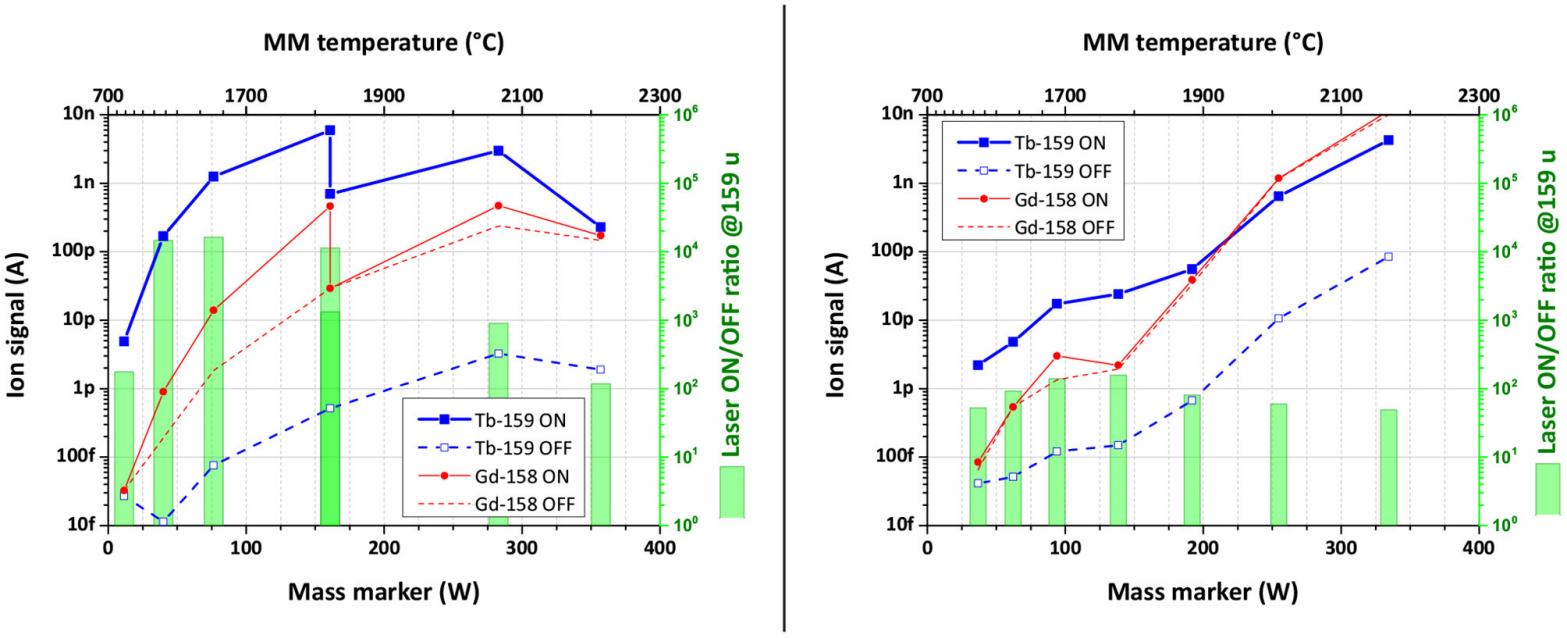
Ion signal behavior depending on the heating power of the mass marker for samples with Gd:Tb ratio of 50:1 **(left)** and 100:1 **(right)**. The ion signal uncertainty of data points is <0.25%.

The performance of the laser ion source in the presence of Gd surplus was studied using the Tb laser resonance ionization scheme B. The results are presented in Figure 7. Both graphs represent the ion signal behavior on mass 159 u (Tb-159, 100% abundance) and on mass 158 u (Gd-158, 25% abundance) depending on the mass marker heating power (temperature) for samples with Gd:Tb ratio of 50:1 (left) and 100:1 (right graph). To observe the influence of the chosen Tb laser ionization scheme, the ion current was measured with lasers switched ON (solid curves) and OFF (dashed curves), representing pure surface ionization for both elements. The ratio between Laser ON/OFF ion signals for Tb gives then the laser enhancement factor (represented with green bars).

One can see from Figure 7 (left), that the observed laser enhancement factor of Tb ion signal in the case of 50:1 Gd:Tb mixture remained the same as for pure Tb sample (1,000 or better). On the other hand, the Gd ion signal was affected by laser beams: in their presence (laser ON) it was 10 times higher than for blocked lasers (laser OFF, see Figure 7). To compensate this finding, the laser power of both transitions was one-by-one reduced to a level, when Gd ion signals with or without lasers are equal (see Figure 7, left, at 1,800°C).

**Figure 8:**
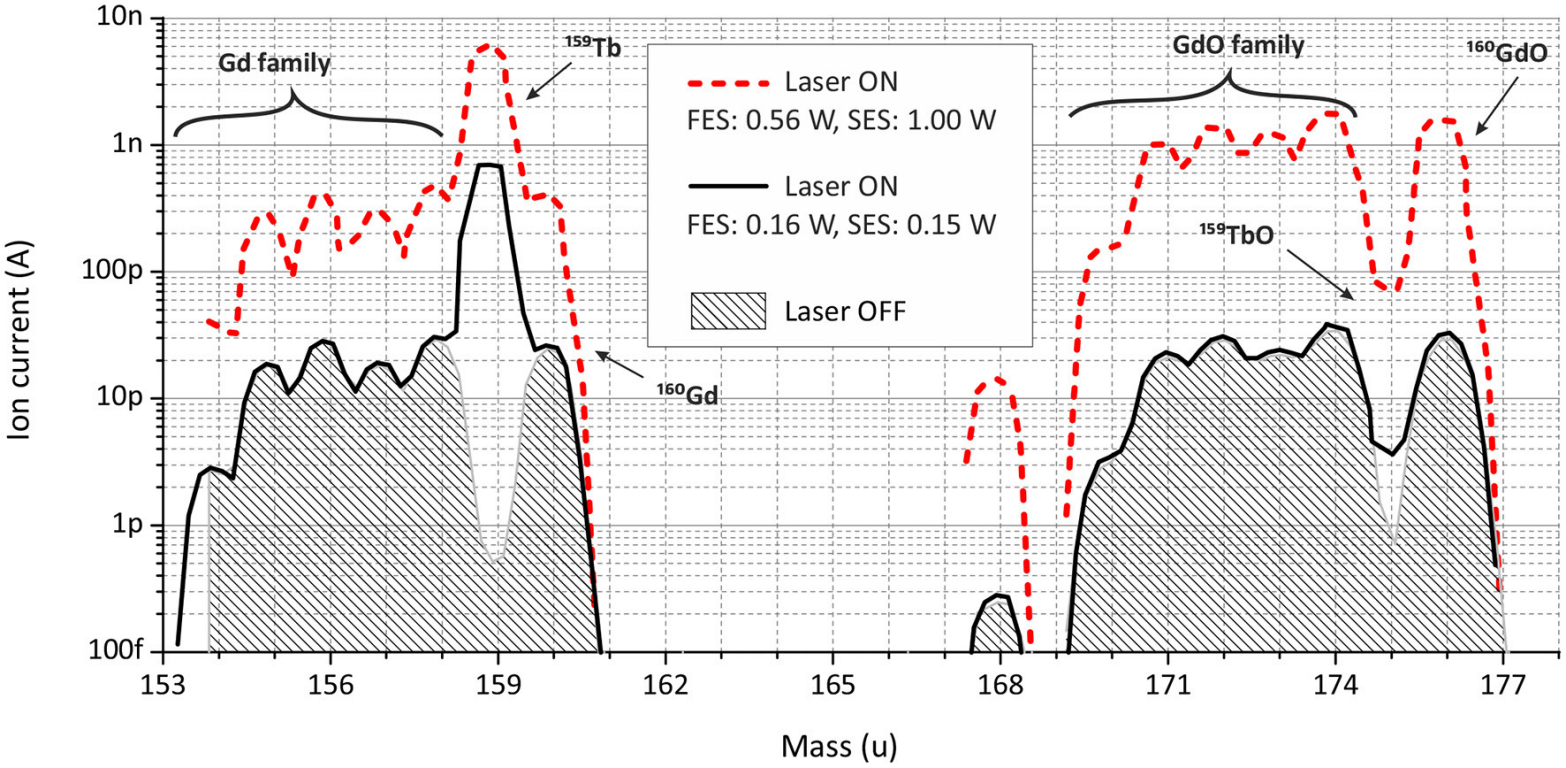
Mass scans in terbium and terbium (II) oxide regions for a sample with a Gd:Tb ratio of 50:1 at the MM temperature around 1,800°C.

The mass scan at 1,800°C demonstrates this effect (Figure 8). The gray filled area represents the ion signal from thermal surface ionization (laser blocked, OFF); the red dashed curve depicts the ion signal in the presence of both lasers on maximal power (laser ON). The reduction of the laser power for the second excitation step (from 1,000 to 154 mW) did significantly change neither Tb nor Gd ion signal for all its isotopes. Reducing additionally the laser power for the first excitation step (from 560 to 160 mW), the ion signal of Gd atoms was decreased to the level of surface ionization (the black solid curve, laser ON). Surely, the Tb ion signal dropped as well, but the enhancement factor was still at the same level as with pure Tb sample. From the literature it is known that for Gd there is a transition from the ground state to an excited level on 23103.7 cm^−1^. Albeit it is 4 cm^−1^ away from FES of scheme B for Tb (23107.3), they are still quite close to each other, and tails of Gd resonance can be affected by the laser power broadening or/and by the Doppler broadening, as laser beams are collinear to the ion beam.

The experiment with 100:1 Gd:Tb ratio sample showed a clear decrease in the Tb laser enhancement factor down to values around 100 (Figure 7, right). The laser power was kept from the start on low values to avoid any contribution to Gd ion signal. At higher temperatures with increasing surface ionization, the ion signals on masses 158 and 159 u became comparable. The influence of neighboring masses on the Tb ion signal did not exceed 10% of the signal at the highest temperature (4% for 50:1 ratio sample). Nevertheless, the Tb signal enhancement induced by laser ionization can be even higher under these unfavorable conditions, but it cannot be observed due to the interference from tails of neighboring mass peaks.

## 4. Discussion and Conclusions

The performed study serve as a basis for successful application of the resonance photoionization of Tb at the MEDICIS laser ion source MELISSA or elsewhere. The newly identified excitation scheme B (Figure 2) can be directly applied for generation of highly intense Tb ion beams due to its excellent ionization efficiency. The obtained value of 53% ionization efficiency is comparable with previous studies of laser ionization on other lanthanides (23, 35). This ionization scheme is currently in use at the CERN-MEDICIS facility to increase the production rate of Tb medical radioisotopes [the efficiency of surface ion source in case of Tb separation is estimated to be about 2% (2)].

Despite a relatively lower efficiency, the single-color two-step laser ionization scheme A (Figure 2) is favorable for specific applications due to the handling of only one laser beam. For example, scheme A was used to start the laser ion source MELISSA in 2019 (37); its simplicity allows to exploit it as a reference or signal control tool during mass separator alignment and optimization. In general, using a set of automated grating-tuned Ti:Sapphire lasers, several pre-defined single-color or two-color two-step schemes can be combined for a rapid and automated switching between ionization of different elements, allowing for sequential extraction of multiple specifically chosen isotopes from the same target.

Further important information can be perceived from laser ion source performance studies in the presence of contaminants. In the case of Tb radioisotope production at CERN-MEDICIS, Gd atoms from a target material, irradiated at an external cyclotron facility, will introduce a dominant contamination. Experimental results with scheme B showed that already the atomic ratio of Gd:Tb of 100:1 will reduce the laser enhancement factor for stable Tb ion signal by an order of magnitude, compared to 50:1 ratio. For RISIKO mass separator, it is caused by the increasing surface ions contribution from tails of adjacent mass peaks of Gd isotopes, which could not be suppressed by the dipole magnet. Another possible reason is a space-charge effect caused by the excess of ions in the hot cavity, which reduces the laser ion source performance; but this is usually observed at much more higher ion currents. Anyway, at 100:1 ratio the extraction of Tb radioisotopes will be technically interfered by Gd isobars, because the surface ion signal of Gd-158 became comparable to Tb-159 laser ion signal.

Obviously, radiochemistry measures are still necessary before and after mass separation to remove the bulk quantity of the target material and to get rid of final traces in the desired product. Here more tests are required with a real target unit to prove these conclusions *in situ*. To elucidate possible interferences from Gd atomic structure, further spectroscopic investigations on Gd atoms have to be undertaken. Eventually, the real production tests at MEDICIS will help to find a balance between the separation efficiency, the ion beam purity and the time consumption for all additional radiochemistry procedures.

## Author's Note

The research was performed in the framework of the European MEDICIS-Promed Network, for which in the contract the IP and conflict of interest parts are well written and defined. AAA was a member of this network, therefore all previous agreements are valid for Novartis as well.

## Data Availability Statement

The original contributions presented in the study are included in the article/Supplementary Material, further inquiries can be directed to the corresponding author/s.

## Author Contributions

VG prepared the first version of the manuscript and is one of those who carried out presented experiments. RF took a part in the manuscript preparation and is one of those who carried out presented experiments. FH took a part in the manuscript preparation, proposed some ideas to experiments, and he is the supervisor of RF. RH, DS, and FW took a part in the manuscript preparation and helped with the realization of experiments. TS is a principal investigator of CERN-MEDICIS project and he took a part in the manuscript preparation. KW took a part in the manuscript preparation, and he is the main supervisor of presented experiments. All authors contributed to the article and approved the submitted version.

## Funding

This research project has been supported by a Marie Skłodowska-Curie Innovative Training Network Fellowship of the European Commission's Horizon 2020 Programme under contract number 642889 MEDICIS-PROMED; by the German Federal Ministry of Education and Research under the consecutive projects 05P12UMCIA and 05P15UMCIA. It has been also partially supported by Equipex ARRONAX-Plus (ANR-11-EQPX-0004), Labex IRON (ANR-11-LABX-18-01), ISITE NExT (ANR-16-IDEX-0007).

## Conflict of Interest

The authors declare that the research was conducted in the absence of any commercial or financial relationships that could be construed as a potential conflict of interest. The handling editor declared a past co-authorship with one of the authors TS.

## Publisher's Note

All claims expressed in this article are solely those of the authors and do not necessarily represent those of their affiliated organizations, or those of the publisher, the editors and the reviewers. Any product that may be evaluated in this article, or claim that may be made by its manufacturer, is not guaranteed or endorsed by the publisher.

## Footnotes

^1^Terbium AAS Standard Solution, AlfaAesar, https://www.alfa.com/en/catalog/088110/.

^2^Terbium AAS Standard Solution, AlfaAesar, https://www.alfa.com/en/catalog/088110/.

^3^Terbium AAS Standard Solution, AlfaAesar, https://www.alfa.com/en/catalog/088110/.

^4^Gadolinium AAS Standard Solution, AlfaAesar, https://www.alfa.com/de/catalog/088065/.
