# Supplementary figures and images for: Terbium Medical Radioisotope Production: Laser Resonance Ionization Scheme Development
Source: Front Med (Lausanne). 2021 Oct 12;8:727557. doi: 10.3389/fmed.2021.727557 (PMC8546115; doi:10.3389/fmed.2021.727557)

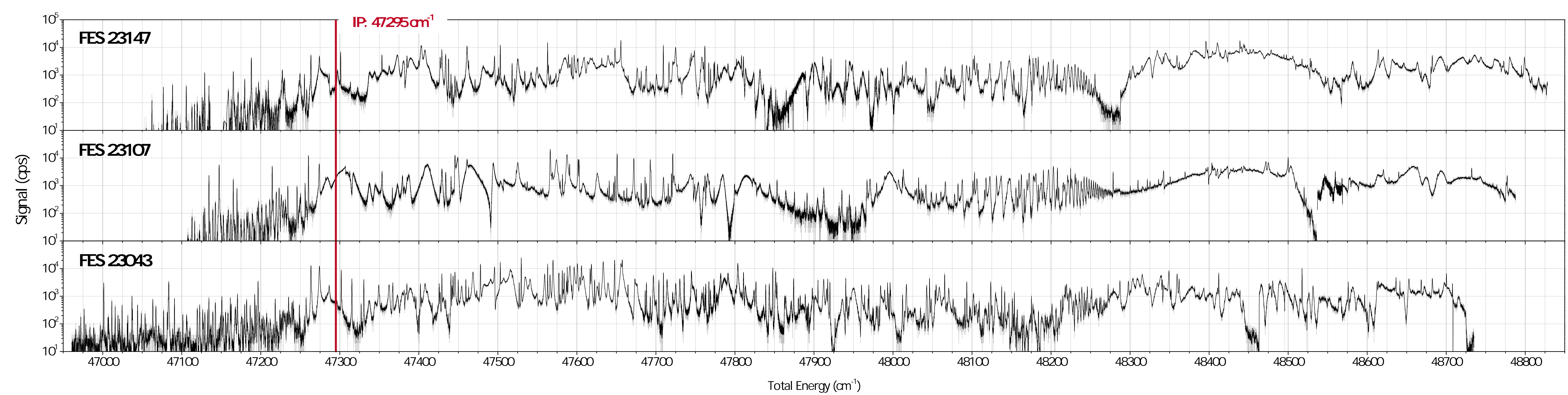

Supplement: Supplementary file 6 [file Image_1.PDF]

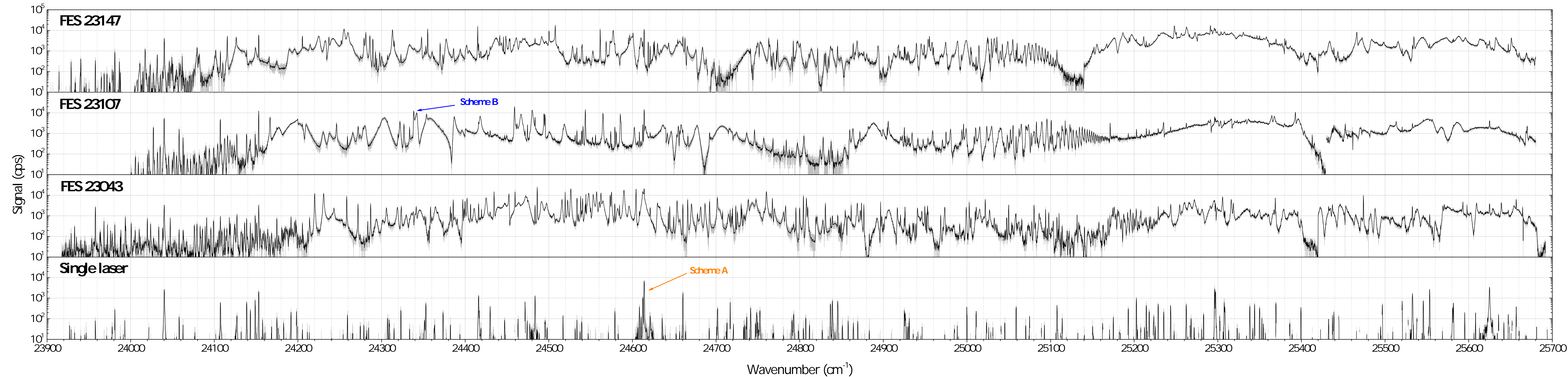

Supplement: Supplementary file 7 [file Image_2.PDF]
