# Supplementary material for: Terbium Medical Radioisotope Production: Laser Resonance Ionization Scheme Development
Source: Front Med (Lausanne). 2021 Oct 12;8:727557. doi: 10.3389/fmed.2021.727557 (PMC8546115; doi:10.3389/fmed.2021.727557)
Supplement: Supplementary file 8 [file Data_Sheet_1.DOCX]

Supplementary Material

# Laser resonance ionization spectroscopy


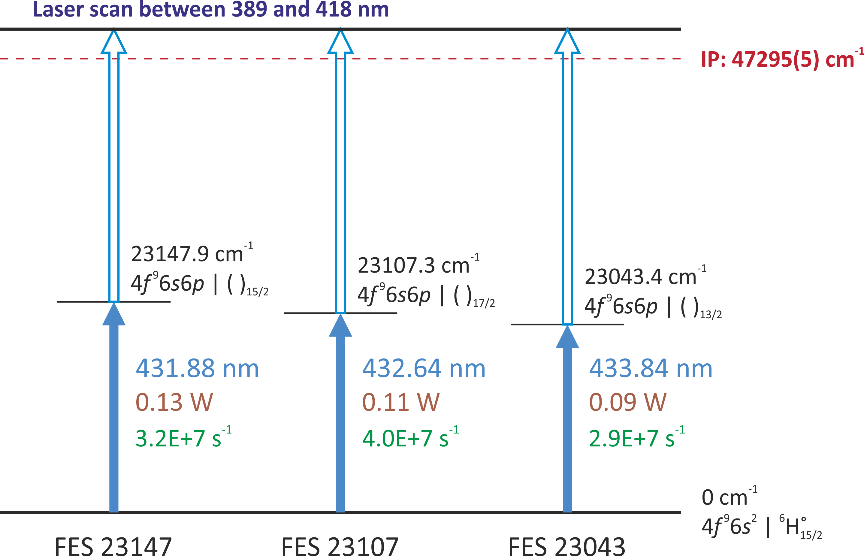


Fig. 1. An overview of investigated first excitation steps (FES) of Tb atoms.

All spectroscopic measurements were accomplished using the wavelength meter of High Finesse (Angstrom) WS6-600, which recorded the fundamental frequency of the grating-tuned Ti:Sapphire laser. The absolute accuracy of the wavemeter is 600 MHz (or 0.02 cm^-1^). The second harmonic was generated externally via a non-linear BBO crystal; the laser scans in SHG region were carried out with manual phase-matching adjustment, tilting the BBO, while spatial beam walk-off was compensated using a system of motorized mirrors and position-sensitive detectors (TEM Aligna Beamlock 2D). To avoid a systematic drift due to the non-ideally synchronized data acquisition, laser scans were performed in both directions, upwards and downwards, with a relatively slow scanning speed; merging both scans, the systematic effects are expected to be below the statistical uncertainty [1]. The position of observed resonances in the spectroscopic measurements was determined approximately to the center of a combined peak, to a data point with the highest ion signal. Statistically, a typical deviation between the centers of the same peak in downwards and upwards scans was within 0.05 cm^-1^. Taking this value as a prudent estimation of the random error and adding to it the absolute accuracy of the wavemeter, the uncertainty of the peak position can be expected at around 0.07 cm^-1^ (for comparison, the minimum difference between two clearly identified peaks in one scan was 0.14 cm^-1^), or for second harmonic of used lasers – 0.14 cm^-1^.

In Fig. 2 of Supplementary Materials, the spectra of observed laser resonances from scanning the second excitation step (SES) in a two-step photoionization process of terbium atoms are presented: starting with first excitation steps (FES) of 23147.9 cm^-1^, of 23107.3 cm^-1^, and of 23043.4 cm^-1^ (see Fig. 1 of Supplementary Materials); as well as a spectrum of resonances observed with a scan of single colour photoionization process.


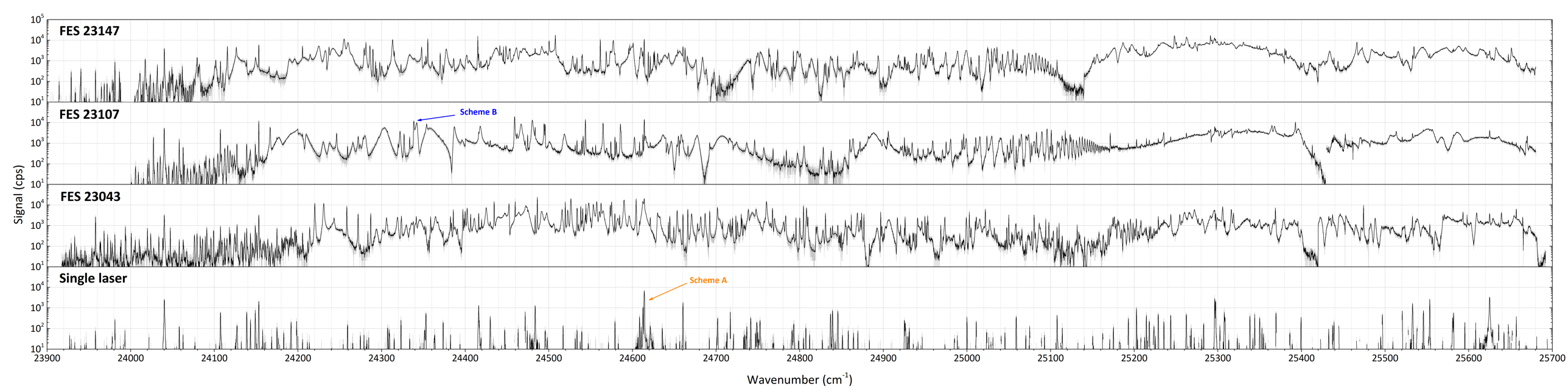


Fig. 2. Spectra of observed resonances of Tb atoms, obtained from scanning the second excitation step for FES 23147, for FES 23107 and for FES 23043, as well as with a single laser scan. On spectra the positions of resonances corresponding to Scheme A and Scheme B (see the main text of the article) are marked.
A high resolution picture can be found in a separate file:

[gvm-FrontMed2021-SupplMat-Figure-2-Laser-Spectroscopy-Spectra.pdf]

In Table 1 of Supplementary Materials, the list of observed single-colour resonances is given. For some lines a probable assignment to known Tb energy levels from NIST Atomic Spectra Database [2] is presented. The energy uncertainty of resonances can be estimated at 0.14 cm^-1^, as it was defined above.

For Table 1 of Supplementary Materials please see a separate file:

[gvm-FrontMed2021-SupplMat-Table-1-single-laser-resonances.xlsx]

In Table 2 of Supplementary Materials, the list of observed resonances towards highly excited and auto-ionizing states (AIS) of Tb atoms via a two-step photoionization process (second excitation steps) is given. The lines, which can be observed only with single laser excitation, are additionally indicated. For each resonance the total energy of the corresponding two-step photoionization process is calculated using the optimized FES energy, measured during the experiment (see Fig. 3 of Supplementary Material). The energy uncertainty of resonances can be estimated at 0.14 cm^-1^, as it was defined above. The uncertainty of the total energy calculation can be prudently evaluated as twice bigger, namely 0.28 cm^-1^.

For Table 2 of Supplementary Materials please see a separate file:

[gvm-FrontMed2021-SupplMat-Table-2-SES-spectra.xlsx]


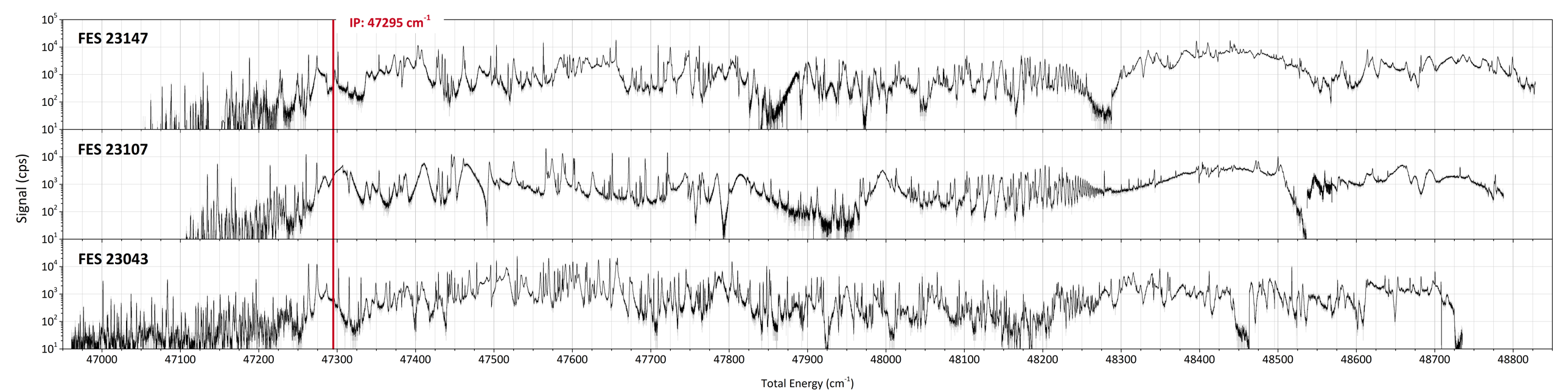


Fig. 3. Spectra of highly-excited and auto-ionizing states of Tb atoms, obtained from scanning the second excitation step for FES 23147, for FES 23107 and for FES 23043.
A high resolution picture can be found in a separate file:

[gvm-FrontMed2021-SupplMat-Figure-3-Odd-high-lying-states-and-AIS-spectra.pdf]

In Table 3 of Supplementary Materials, the list of possible odd auto-ionizing states is given. For each state the J-value assignment is proposed based on the selection rules for E1 transitions. The uncertainty of AIS position from this work can be estimated at 0.28 cm^-1^ from the total energy calculation uncertainty; the position discrepancy of AIS, accessible from different FES, is lower and, in average, about 0.16 cm^-1^. To compare the position of odd AIS, the results from [3] are included in the table. The uncertainty of AIS position from [3] is around 3 cm^‑1^.

For Table 3 of Supplementary Materials please see a separate file:

[gvm-FrontMed2021-SupplMat-Table-3-Odd-AIS.xlsx]


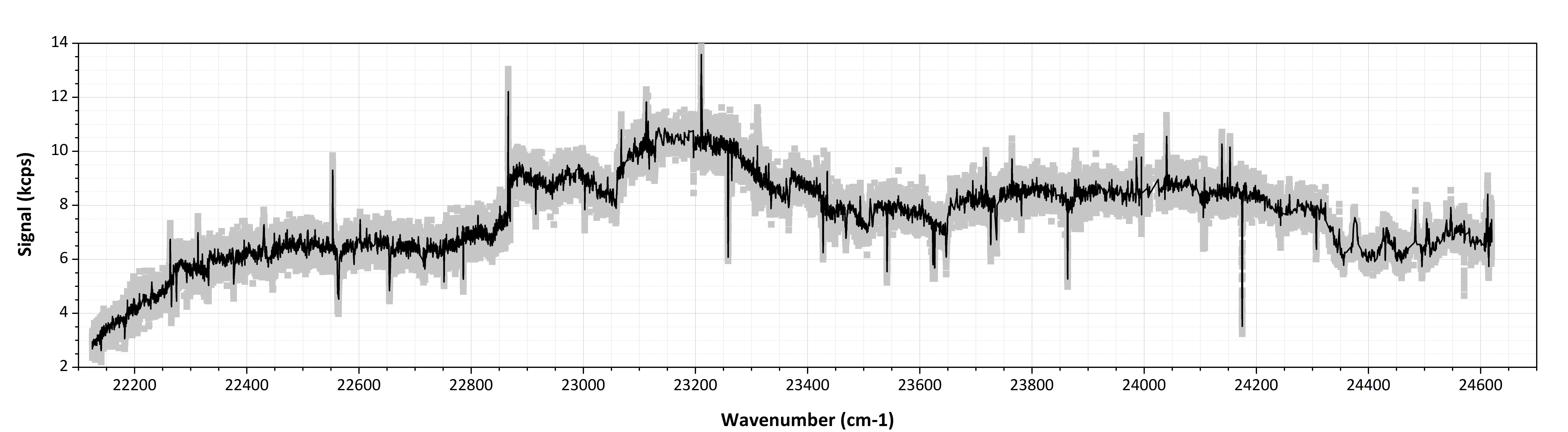


Fig. 4. Spectrum of «dip» and «peak» resonances obtained from scanning the second laser with a first laser tuned to 24614.06 cm-1.

In Fig. 4 of Supplementary Materials, one can see a spectrum of resonances obtained with a system of two lasers: one was fixed on the most intensive single colour resonance (24614), the working energy of another one was tuned downwards to a lower energy region below the resonance (24614), trying to find out possible even auto-ionizing states of Tb atoms with the resonance (24614) as a first excitation step. The measurement was only partly accomplished, and thus only the most meaningful results are presented. In Table 4 of Supplementary Materials, the list of observed in this measurement «dip» and «peak» resonances is given, as well as a probable assignment to known Tb energy levels from NIST Atomic Spectra Database [2]. The uncertainty of observed resonances can be estimated at 0.14 cm^-1^, as it was defined before.

For Table 4 of Supplementary Materials please see a separate file:

[gvm-FrontMed2021-SupplMat-Table-4-Dips-and-AIs.xlsx]

In Table 5 of Supplementary Materials, the list of possible even auto-ionizing states is given: the majority of AIS was taken from previous studies [3], [4], [5], where a three-step resonance photoionization process to even AIS was considered. The new levels from this work are proposed based on the high intensity of observed single laser resonances (which could serve as a sign of a transition to a close lying AIS) or based on their location nearby the known AIS from literature. Where it was possible, the J-value assignment for AIS is proposed based on the selection rules for E1 transitions. This is not the full spectrum; only clearly observable peaks from other works are presented.

The uncertainty of AIS position from [3] is 3 cm-1. The uncertainty of AIS position from [4], [5] was estimated at 0.1 cm^-1^. The uncertainty of AIS position from this work can be estimated at 0.28 cm^‑1^ from the total energy calculation uncertainty.

For Table 5 of Supplementary Materials please see a separate file:

[gvm-FrontMed2021-SupplMat-Table-5-Possible-even-AIS.xlsx]

# Characterization of the most efficient laser ionization

From the spectroscopic results, several highly intensive second excitation transitions to auto-ionizing states were found. To choose probably the most efficient two-step resonance ionization scheme, these SES were compared between each other under more less similar conditions: the laser power for each FES was kept constant (102 mW) as well as the laser power for SES (102 mW) to minimize power-related effects. The full comparison was accomplished during one day in one measurement with one sample. To ensure the presence of a proper amount of sample material in the hot cavity during the measurement, the most intensive single colour resonance (at 2x 12307.07 cm^-1^) was used as a reference signal (see Fig. 5 of Supplementary Materials). Due to small oscillations of the reference signal, it was normalized to 8000 cps with the appropriate correction of results of the comparison. The characteristics of the most intensive schemes, marked with a number on Fig. 5 (which signal was higher than the reference one), are presented in Fig. 6 of Supplementary Materials.


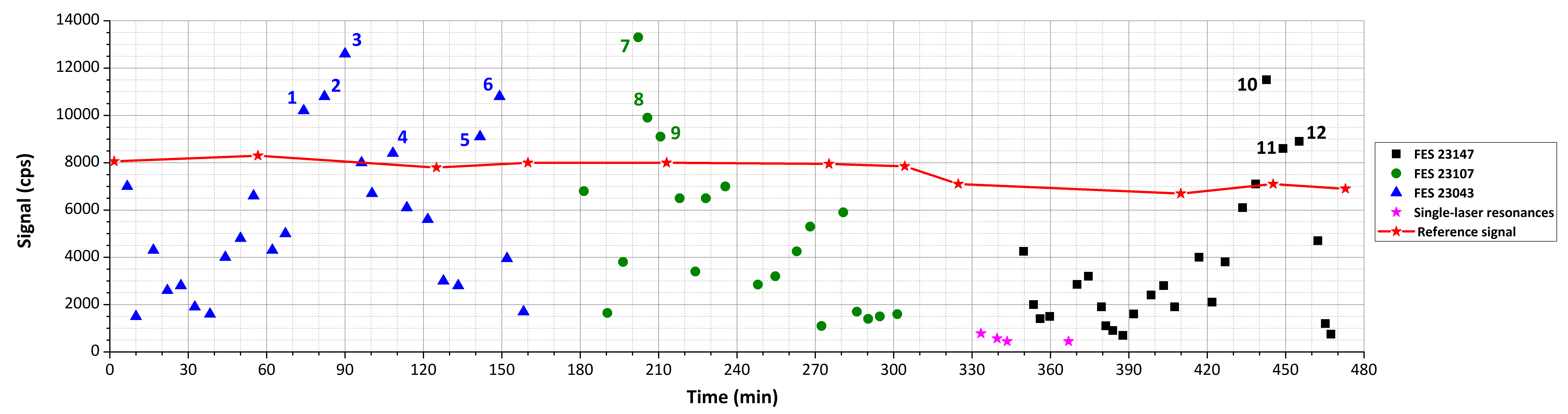


Fig. 5. An overview of the comparison measurement of different two-step resonance ionization schemes for Tb.

Fig. 6. The list of two-step photoionization schemes for FES 23043, FES 23107, FES 23147, compared between each other under similar conditions and equal laser output power for both steps, as well as with the reference signal from the single-colour two-step photoionization scheme 24614.

From the comparison of different two-step resonance ionization processes, one scheme for each first excitation step with the highest intensity of the signal was chosen: #3 of FES 23043, #7 of FES 23107 and #10 of FES 23147 (see Fig. 6). To characterize these schemes and the highly intensive single-colour resonance, the saturation measurements were undertaken. The results can be found in Fig. 7 - Fig. 10 of Supplementary Materials. As one can see from graphs, all FES were well-saturated, except the single-colour two-step photoionization scheme, as it requires a higher laser output power to be saturated (almost 100 mW saturation power compared to a couple of mW for other schemes). All SES demonstrated a clear linear trend in the saturation curve. This is a common behaviour of an ionization step, which requires as much power as possible to get more intensive signal (the same as for single-colour two step scheme, as it combines in itself both FES and SES). For further investigations (efficiency measurements etc., see the main text of article), the scheme #7 of FES 23107 was chosen, because it provides the highest ion signal among all others with the same laser output power, what may serve as a sign of the most efficient ionization process for Tb.


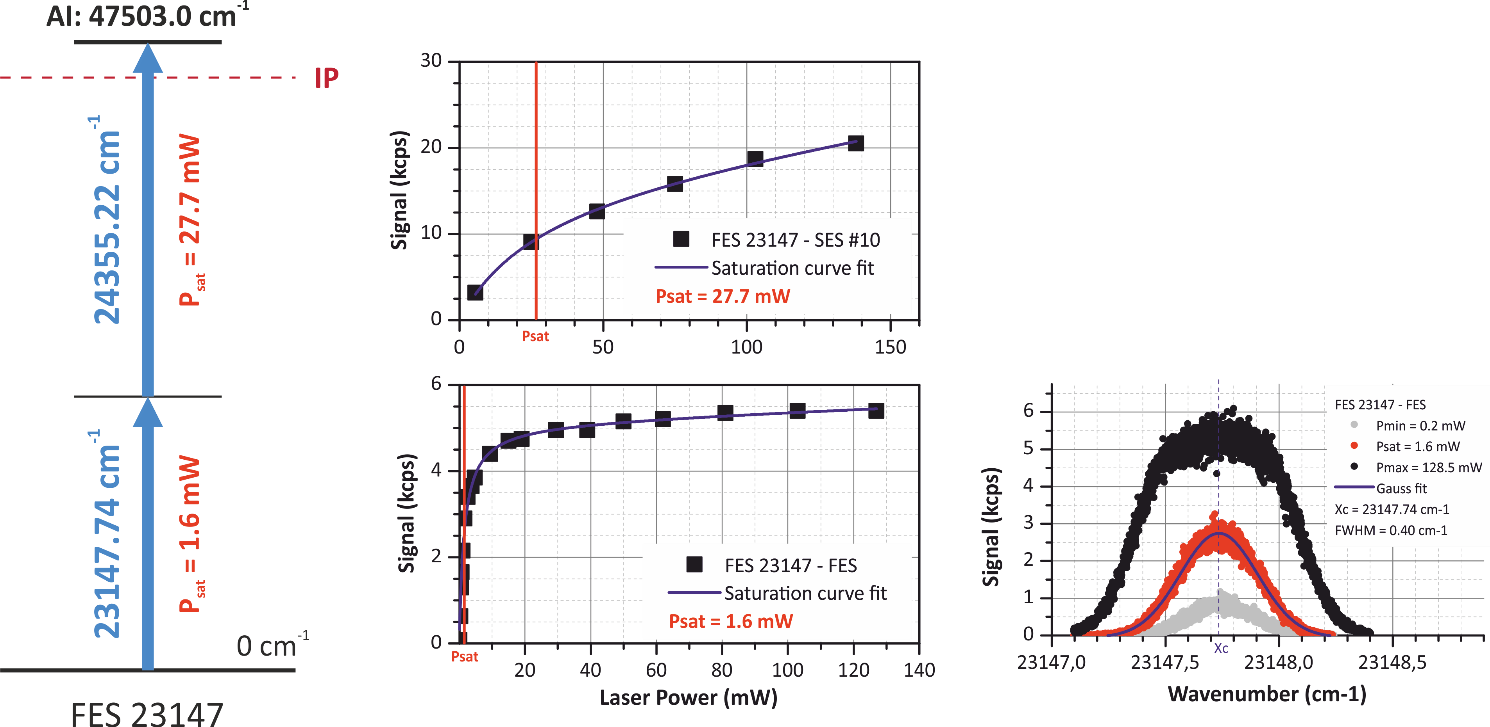


Fig. 7. The saturation behavior of Tb two-step photoionization scheme with FES 23147, and the most intensive corresponding SES transition to the AIS on 47503.0 cm^-1^.


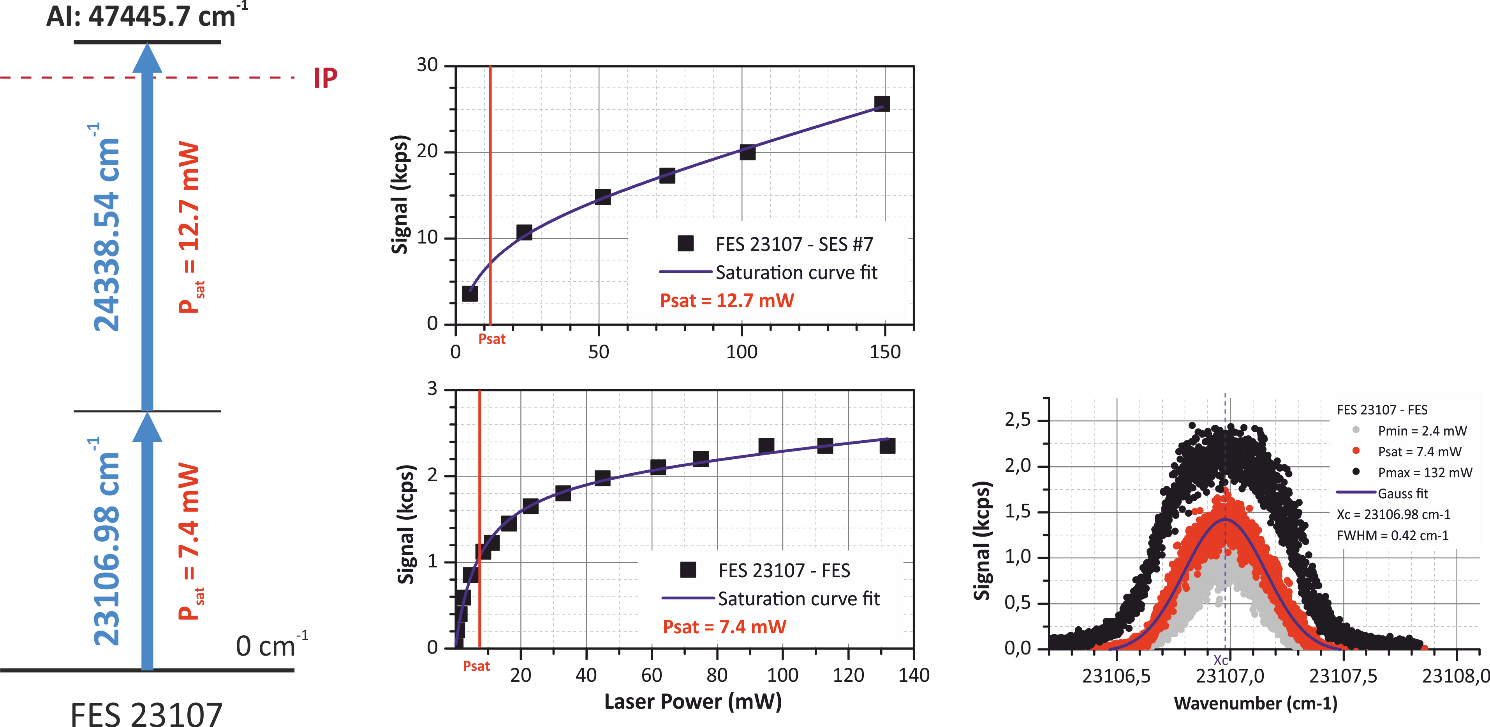


Fig. 8. The saturation behavior of Tb two-step photoionization scheme with FES 23107, and the most intensive corresponding SES transition to the AIS on 47445.7 cm^-1^
(denoted in the article as Scheme B).


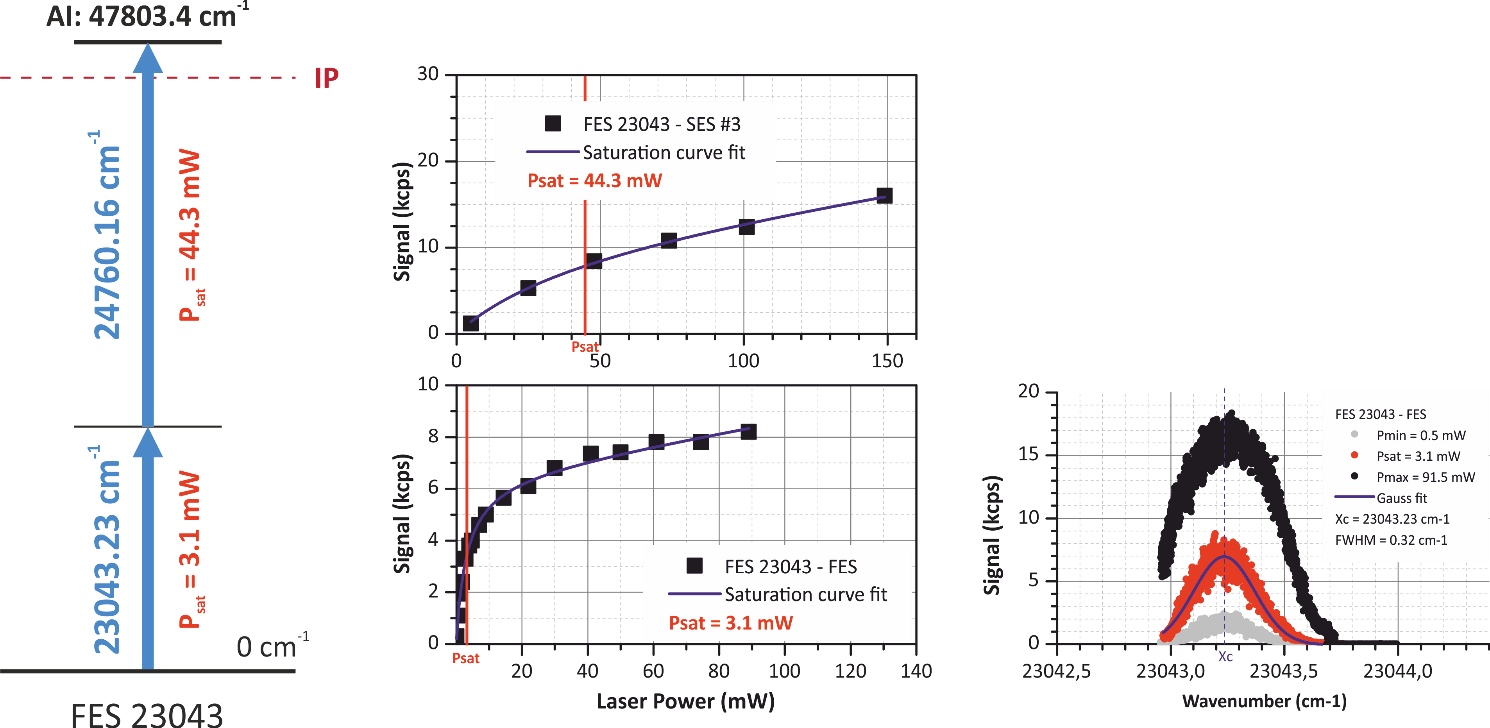


Fig. 9. The saturation behavior of Tb two-step photoionization scheme with FES 23043, and the most intensive corresponding SES transition to the AIS on 47803.4 cm^-1^.


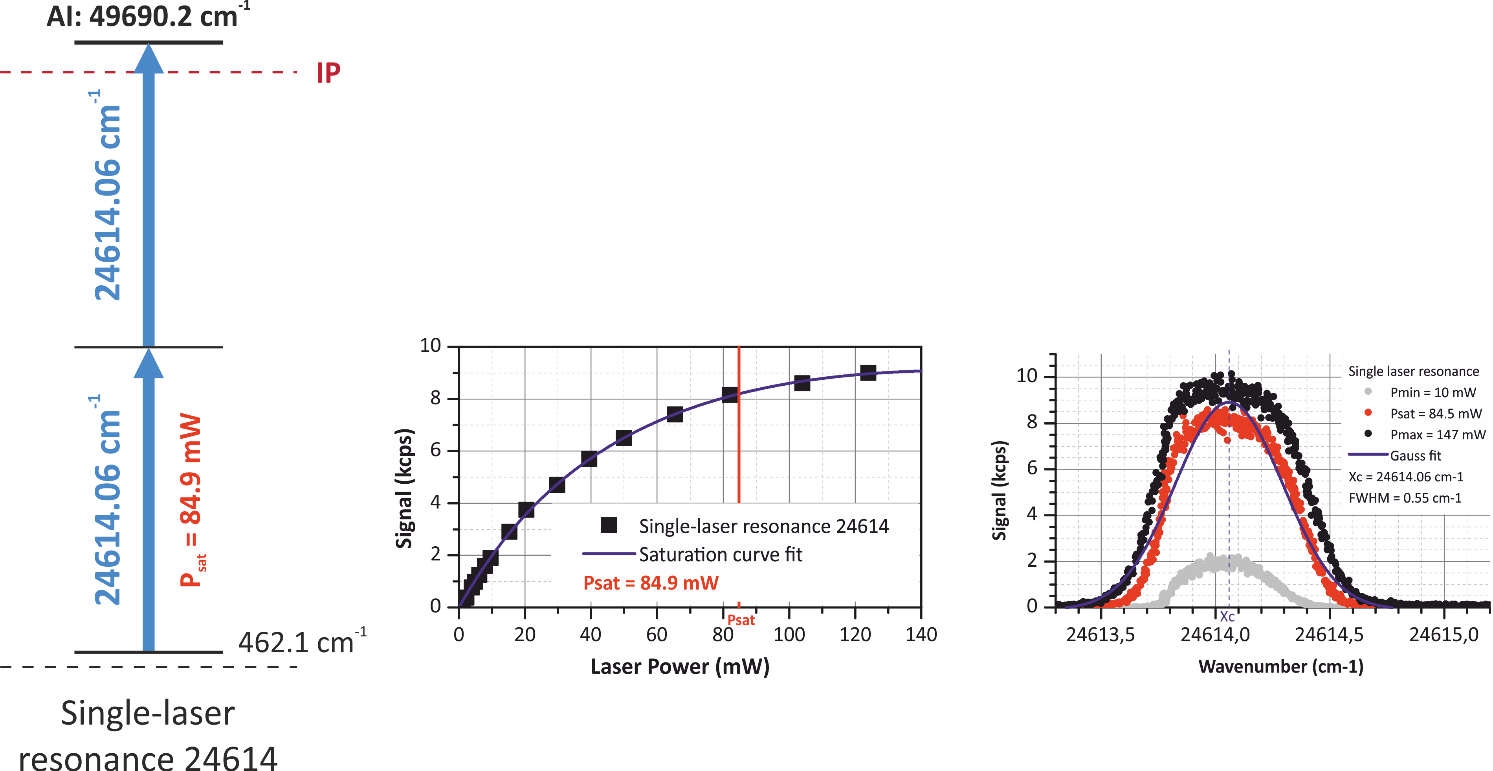


Fig. 10. The saturation behavior of Tb two-step single-colour photoionization scheme (24614) from a thermally populated energy level on 462.1 cm^-1^ towards a probable AIS on 49690.2 cm^-1^ (denoted in the article as Scheme A).

# References

[1] D. Studer *et al.*, “Atomic transitions and the first ionization potential of promethium determined by laser spectroscopy,” *Phys. Rev. A*, vol. 99, no. 6, p. 062513, Jun. 2019, doi: 10.1103/PhysRevA.99.062513.

[2] J. Ralchenko, Y. and Kramida, A.E. and Reader, “NIST Atomic Spectra Database (ver. 5.7.1),” 2019. https://www.nist.gov/pml/atomic-spectra-database (accessed Dec. 15, 2019).

[3] V. N. Fedoseev, V. I. Mishin, D. S. Vedeneev, and A. D. Zuzikov, “Laser resonant photoionization spectroscopy of highly excited and autoionization states of terbium atoms,” *J. Phys. B At. Mol. Opt. Phys.*, vol. 24, no. 7, pp. 1575–1583, Apr. 1991, doi: 10.1088/0953-4075/24/7/012.

[4] T. Gottwald, J. Lassen, Y. Liu, C. Mattolat, S. Raeder, and K. Wendt, “Laser Resonance Ionization Spectroscopy of the Lanthanides Tb , Dy and Ho as Homologues to Actinides and Super Heavy Elements,” *AIP Conf. Proc.*, vol. 1104, pp. 138–143, 2009, doi: 10.1063/1.3115590.

[5] T. Gottwald, “Studium hochkomplexer atomarer Spektren mittels Methoden der Laserresonanzionisation,” Johannes Gutenberg University Mainz, 2011.
